# Efficacy and safety of perioperative immunotherapy for locally advanced thymic squamous cell carcinoma: a retrospective pilot study

**DOI:** 10.3389/fsurg.2025.1722026

**Published:** 2025-12-15

**Authors:** Dong Li, Yingbo Zou

**Affiliations:** Department of Thoracic Surgery, The Third Affiliated Hospital of Chongqing Medical University, Chongqing, China

**Keywords:** thymic squamous cell carcinoma, perioperative immunotherapy, PD-1 inhibitors, efficacy, treatment-related adverse reactions

## Abstract

**Background:**

Thymic squamous cell carcinoma (TSCC), the predominant subtype of thymic carcinoma, is a rare and aggressive malignancy. Although the clinical benefits of perioperative immunochemotherapy for non-small cell lung cancer have been confirmed, its role in TSCC remains unclear. This study was performed to evaluate the efficacy and safety of perioperative immunotherapy for locally advanced TSCC.

**Methods:**

The clinical data of 10 locally advanced TSCC patients treated with perioperative immunotherapy were retrospectively analyzed. All the patients received neoadjuvant PD-1 inhibitors plus platinum-based chemotherapy, followed by surgery and adjuvant immunotherapy. Surgical and pathological outcomes, postoperative complications, treatment-related adverse events (TRAEs), and survival outcomes were all assessed.

**Results:**

After neoadjuvant immunotherapy, 60% (6/10) of patients achieved partial response and 40% (4/10) obtained stable disease, with the objective response rate of 60% and disease control rate of 100%. R0 resection was achieved in 80% (8/10) of patients, with 2 achieving complete pathological response. All the patients experienced at least one grade 1–2 TRAEs, but no grade 3–4 TRAEs occurred. The most commonly TRAEs were anorexia (70%) and alopecia (70%), followed by fatigue (60%). During the follow-up of 30 months, only 2 patients were dead, with recurrence-free survival of 17 and 19 months and overall survival of 23 months for both.

**Conclusion:**

Perioperative immunotherapy exhibits a promising resectable rate in locally advanced TSCC, with a manageable safety profile, although survival benefits have yet to be established. In the future, a prospective randomized controlled trial should be performed to further clarify the role of perioperative immunotherapy for locally advanced TSCC.

## 1 Introduction

Thymic squamous cell carcinoma (TSCC) is a rare and aggressive subtype of thymic epithelial malignancies, representing 15%–20% of all thymic cancers (1–3). Characterized by marked aggressiveness, TSCC is typically diagnosed at advanced stages (III or IV), significantly limiting opportunities for radical surgery (4–6). Currently, achieving microscopically negative margins (R0 resection) remains challenging in clinic, even with multimodal therapeutic approaches (7). The 5-year survival rate is below 30% for unresectable cases, underscoring the imperative for innovative therapeutic strategies (4, 8–10).

The current treatment modalities for TSCC primarily encompass surgical resection, chemotherapy, and radiotherapy (11–13). However, the dual challenges of late-stage presentation and inherent resistance to conventional therapies result in suboptimal treatment responses and frequent disease recurrence (14, 15). While immune checkpoint inhibitors (ICIs), particularly anti-PD-1 antibodies, have demonstrated transformative efficacy across multiple malignancies (16, 17), their therapeutic potential in TSCC, especially in the perioperative context, is not fully understood.

In recent years, perioperative immunotherapy, incorporating both neoadjuvant and adjuvant modalities, has shown promise for improving surgical outcomes and prolonging survival in non-small cell lung cancer (NSCLC) (18, 19). Neoadjuvant immunotherapy may facilitate tumor downstaging to enhance resectability while initiating systemic immune activation. Adjuvant administration could target micrometastatic disease and establish durable immunological memory. However, there are few studies on the application of perioperative immunotherapy in TSCC. Our previous study attempted to evaluate the role of immunotherapy in advanced/metastatic TSCC settings (20), but the robust data regarding the perioperative application were still scarce. Several studies have not meticulously distinguished the pathological subtypes of thymic carcinoma, and some even include type B3 thymomas, potentially increasing the risk of immune-related adverse events (irAEs) (21–23).

Given the aggressive biology of TSCC and its limited response to traditional therapies, it is quite necessary to explore the role of perioperative immunotherapy in TSCC. Herein, this study was performed to systematically evaluate the clinical efficacy and safety of perioperative immunotherapy in patients with histologically confirmed locally advanced TSCC.

## 2 Materials and methods

### 2.1 Patients

TSCC patients treated with perioperative immunotherapy at The Third Affiliated Hospital of Chongqing Medical University between January 2021 and December 2024 were collected consecutively. Inclusion criteria included: (1) histologically confirmed locally advanced TSCC; (2) receiving perioperative immunotherapy; (3) complete clinical and follow-up records. Patients with concurrent malignancies, active autoimmune disorders, or those failing to complete the scheduled immunotherapy regimen were excluded.

The study protocol was approved by the Institutional Review Board of The Third Affiliated Hospital of Chongqing Medical University (IRB-2021-033) and conducted in accordance with the Declaration of Helsinki. Written informed consent was obtained from all participants or their legally authorized representatives prior to data collection.

### 2.2 Data collection

The data involved in the study were systematically extracted from electronic medical records and operative reports, mainly including patient clinicopathological characteristics, perioperative immunotherapy regimens, surgical and pathological outcomes, as well as treatment-related adverse reactions (TRAEs). The patient clinicopathological characteristics comprised age at diagnosis, gender, Eastern Cooperative Oncology Group Performance Status (ECOG PS) score, histological subtype, maximum tumor diameter measured in the axial plane imaging and tumor staging according to the Masaoka-Koga classification system.

Regarding perioperative immunotherapy, the ICI types and cycle number of the neoadjuvant treatment universally incorporating immunochemotherapy combinations were recorded. Postoperative adjuvant chemotherapy/radiotherapy was determined through multidisciplinary tumor board consensus, guided by individualized clinical-pathological profiles and real-time therapeutic response monitoring.

### 2.3 Surgical procedures and pathological assessment

All surgical procedures were performed by thoracic surgeons with subspecialty expertise in mediastinal oncology, adhering to the principle of achieving complete macroscopic resection (R0) as the primary surgical endpoint. Perioperative parameters encompassing surgical approach (open thoracotomy vs. video-assisted thoracoscopic surgery), surgical duration, intraoperative blood loss, transfusion volume, and postoperative hospital stays were all documented using standardized case report forms. Postoperative complications were systematically classified according to the Clavien-Dindo classification system through daily clinical assessments during hospitalization and subsequent follow-up evaluations.

Histopathological features of the resected specimens were assessed by experienced pathologists, including three-dimensional tumor size, histopathological response evaluated by the tumor regression grading system, and surgical margin status according to the criteria from College of American Pathologists. Pathological complete response (pCR) was defined as no viable tumor cells at the primary tumor and in the lymph nodes, and major pathological response (MPR) was defined as less than 10% of viable tumor cells at the primary tumor site (24). Therapeutic response to neoadjuvant immunotherapy was assessed through Response Evaluation Criteria in Solid Tumors (RECIST) version 1.1 guidelines, and the objective response rate (ORR) and disease control rate (DCR) were calculated, respectively.

TRAEs were graded based on the National Cancer Institute Common Terminology Criteria for Adverse Events (version 5.0). Special attention should be paid to severe irAEs, including myocarditis, pneumonitis, and myasthenia gravis.

### 2.4 Follow-up

Patients were followed up according to the following protocol: quarterly evaluations during the first 2 years, and then biannual assessments. The final follow-up date was March 31, 2025. The surveillance regimen comprised comprehensive physical examinations combined with thoracic computed tomography (CT) imaging. Observational endpoints included recurrence-free survival (RFS) and overall survival (OS).

### 2.5 Statistical analysis

Statistical analyses were conducted using SPSS software (version 29.0; IBM Corp). Continuous variables were presented as either median with interquartile range or mean ± standard deviation (SD), and compared using the Mann–Whitney *U* test or *t* test. Categorical variables were summarized using frequencies and percentages, and compared by the Fisher's exact test. Survival outcomes were analyzed through Kaplan–Meier methods. A two-tailed *p*-value < 0.05 was considered statistically significant.

## 3 Results

### 3.1 Patient baseline characteristics

Between January 2021 and December 2024, there were a total of 22 TSCC patients who received perioperative immunotherapy at The Third Affiliated Hospital of Chongqing Medical University. After excluding 2 cases of completely resected stage I TSCC, 3 cases of active autoimmune diseases and 7 cases of incomplete clinical data, 10 patients including 8 males (8/10, 80%) and 2 females (2/10, 20%) were finally eligible for the study. Notably, type B thymoma was confirmed to be excluded through comprehensive immunohistochemical analysis. The patients had the median age of 53 years (range: 39–67 years) at diagnosis, in which 8 cases were assessed as ECOG PS 0 (8/10, 80%) and 2 were ECOG PS 1 (2/10, 20%). According to the Masaoka-Koga classification system, there were 7 cases (7/10, 70%) at stage IIIB and 3 cases (3/10, 30%) at stage IIIA. The pre-treatment tumor diameter was (59.30 ± 19.40) mm. The baseline characteristics of all patients included in this study are summarized in Table 1.

**Table 1:** The baseline characteristics and treatment of patients included in the study.

No.	Gender	Age, years	ECOG score	NAT regimens	Tumor staging	NAT cycles	Tumor diameter (mm)				Preoperative evaluation	pCR	Postoperative treatment
							Before treatment	After 2 cycles	After 3 cycles	After 4 cycles			
P1	Male	60	0	Pembrolizumab (200 mg, d1), Albumin-bound Paclitaxel (220 mg/m^2^, d2) + Carboplatin (AUC = 5, d2)	IIIB	3	60	51	51	-	SD	No	-
P2	Female	54	0	Pembrolizumab (200 mg, d1), Albumin-bound Paclitaxel (220 mg/m^2^, d2) + Carboplatin (AUC = 5, d2)	IIIB	3	45	34	32	-	PR	No	Pembrolizumab (200 mg, d1), Albumin-bound Paclitaxel (220 mg/m^2^, d2) + Carboplatin (AUC = 5, d2) *3 cycles + Pembrolizumab (200 mg, d1) *12 + mediastinal RT
P3	Male	65	1	Pembrolizumab (200 mg, d1), Albumin-bound Paclitaxel (220 mg/m^2^, d2) + Carboplatin (AUC = 5, d2)	IIIB	3	55	45	43	-	PR	No	Pembrolizumab (200 mg, d1) *12 + mediastinal RT + Pembrolizumab (200 mg, d1), Albumin-bound Paclitaxel (220 mg/m^2^, d2) + Carboplatin (AUC = 5, d2) *3 cycles
P4	Male	50	0	Sintilimab (200 mg, d1), Albumin-bound Paclitaxel (220 mg/m^2^, d2) + Carboplatin (AUC = 5, d2)	IIIB	4	62	52	52	50	SD	No	Sintilimab (200 mg, d1), Albumin-bound Paclitaxel (220 mg/m^2^, d2) + Carboplatin (AUC = 5, d2) *1 cycles
P5	Male	67	1	Sintilimab (200 mg, d1), Albumin-bound Paclitaxel (220 mg/m^2^, d2) + Carboplatin (AUC = 5, d2)	IIIB	3	43	32	33	-	PR	No	Sintilimab (200 mg, d1), Albumin-bound Paclitaxel (220 mg/m^2^, d2) + Carboplatin (AUC = 5, d2) *1 cycles + Sintilimab (200 mg, d1) *12
P6	Male	46	0	Sintilimab (200 mg, d1), Albumin-bound Paclitaxel (220 mg/m^2^, d2) + Carboplatin (AUC = 5, d2)	IIIA	2	27	22	22	-	SD	No	Sintilimab (200 mg, d1), Albumin-bound Paclitaxel (220 mg/m^2^, d2) + Carboplatin (AUC = 5, d2) *2 cycles + Sintilimab (200 mg, d1) *14
P7	Male	47	0	Sintilimab (200 mg, d1), Albumin-bound Paclitaxel (220 mg/m^2^, d2) + Carboplatin (AUC = 5, d2)	IIIA	4	98	70	65	65	PR	No	Sintilimab (200 mg, d1), Albumin-bound Paclitaxel (220 mg/m^2^, d2) + Carboplatin (AUC = 5, d2) *2 cycles + Sintilimab (200 mg, d1) *12
P8	Male	39	0	Sintilimab (200 mg, d1), Albumin-bound Paclitaxel (220 mg/m^2^, d2) + Carboplatin (AUC = 5, d2)	IIIB	4	72	51	44	44	PR	Yes	Sintilimab (200 mg, d1) *4
P9	Male	66	0	Pembrolizumab (200 mg, d1), Albumin-bound Paclitaxel (220 mg/m^2^, d2) + Carboplatin (AUC = 5, d2)	IIIA	4	73	67	67	65	SD	No	-
P10	Female	52	0	Sintilimab (200 mg, d1), Albumin-bound Paclitaxel (220 mg/m^2^, d2) + Carboplatin (AUC = 5, d2)	IIIB	3	58	30	28	-	PR	Yes	Sintilimab (200 mg, d1), Albumin-bound Paclitaxel (220 mg/m^2^, d2) + Carboplatin (AUC = 5, d2) *2 cycles

NAT, neoadjuvant treatment; pCR, pathological complete response; AUC, area under the curve; SD, stable disease; PR, partial response.

### 3.2 Perioperative treatment

The median 3 cycles (range: 2–4) of neoadjuvant immunochemotherapy comprising anti-PD-1 inhibitors were used. Afte 3 cycles of treatment, the tumor diameter was (43.30 ± 14.79) mm, significantly lower than that before treatment (*p* < 0.01). Immunotherapy regimens included pembrolizumab (200 mg, q3w) or sintilimab (200 mg, q3w), combined with nanoparticle albumin-bound paclitaxel (220 mg/m^2^, q3w) and carboplatin (AUC = 5, q3w) (Table 1). 8 patients were treated with adjuvant immunotherapy for the median 8.5 cycles (range: 1–16), while 2 declined the postoperative adjuvant treatment encompassing radiotherapy, chemotherapy, and immunotherapy due to personal reasons. Concurrent adjuvant chemotherapy was administered to 7 patients, with the median 2.0 cycles (range: 1–3), among whom one R1-resected case received consolidative radiotherapy after chemotherapy, and one R2-resected patient with a high PD-L1 tumor proportion score of 95% received PD-1 inhibitor monotherapy but subsequently required salvage chemoimmunotherapy and radiotherapy upon recurrence.

### 3.3 Surgical and pathological outcomes

Among 10 patients receiving perioperative immunotherapy, 6 (6/10, 60%) achieved partial response (PR) and 4 (4/10, 40%) achieved stable disease (SD) after neoadjuvant immunotherapy. The ORR and DCR were 60% and 100%, respectively.

**Figure 1:**
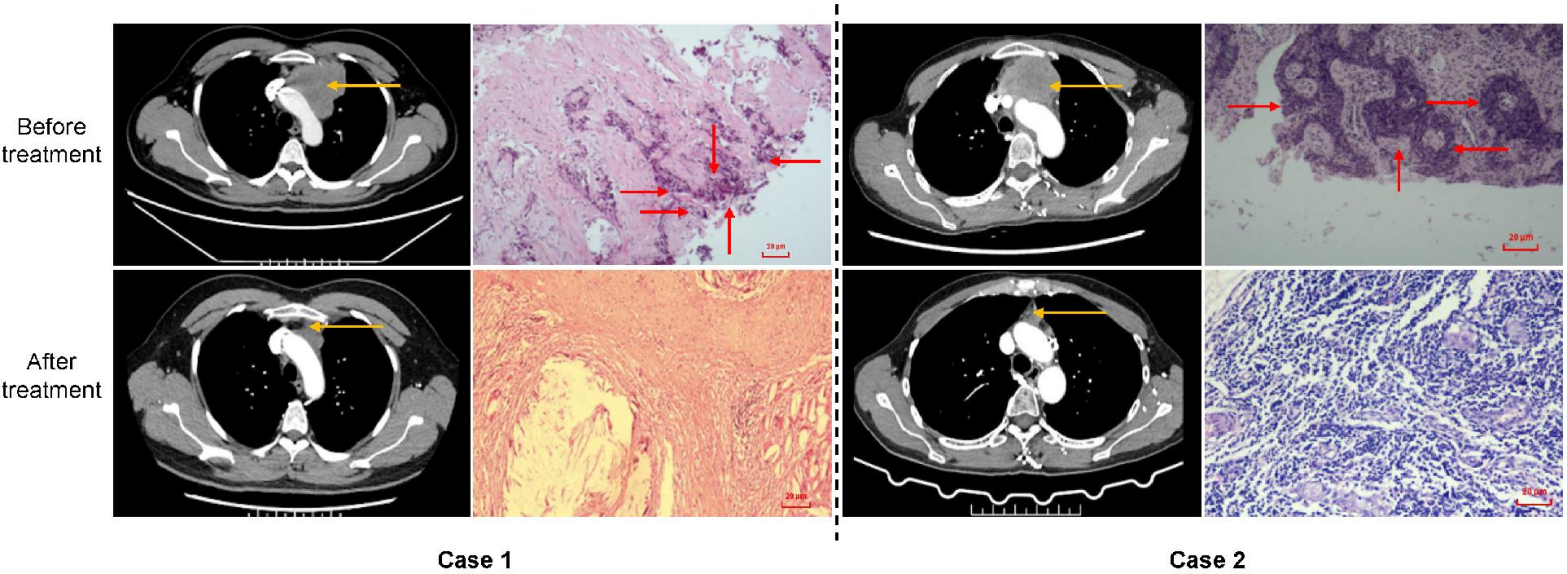
Contrast-enhanced computed tomography and pathological findings of the two patients with pathological complete response. The yellow and red arrows head towards the tumor lesion and tumor cells, respectively. In case 1, the pre-treatment pathological findings showed that the tumor located in the anterior mediastinum was lowly differentiated squamous cell carcinoma. In case 2, the pre-treatment pathological findings revealed that the tumor located in the anterior mediastinum was lowly differentiated squamous cell carcinoma. Postoperative pathological results were presented by hematoxylin and eosin (HE, ×100).

Surgical resection was successfully performed in 10 patients, including R0 in 8 patients (80%), R1 in 1 patient (10%) and R2 in 1 patient (10%) (Table 2). Among R0-resected cases, 5 (5/8, 62.5%) required concomitant vascular resection/reconstruction for tumor involvement, primarily involving the innominate vein and superior vena cava. Pericardial resection (5/8, 62.5%) and pulmonary resection (3/8, 37.5%) were also performed due to adjacent organ invasion. The R1-resected case demonstrated positive resection margins at peritumoral lymph nodes, while the R2-resected case involved the residual tumor adherent to the aortic adventitia after maximal debulking. Pathological assessment of the resected specimens revealed marked reduction in tumor volume and enhanced immune cell infiltration in all PR patients, including 2 cases of pCR (Figure 1).

**Table 2:** Surgical and pathological outcomes and postoperative complications of the patients.

Variables	*n* (%)
Extent of resection	
R0	8 (80.0)
R1	1 (10.0)
R2	1 (10.0)
Median operative time, min	240 (120–345)
Median blood loss, mL	200 (50–700)
Median postoperative hospital stays, days	12.5 (5–20)
Median chest tube duration (days)	5 (3–12)
Pathological evaluation	
pCR	2 (20.0)
MPR	2 (20.0)
Non-pCR/MPR	6 (60.0)
Organ invasion	
Pericardium	5 (50.0)
Brachiocephalic vein	5 (50.0)
Lung	3 (30.0)
Aorta	1 (10.0)
Surgical methods	
VATS/RATS	4 (40.0)
Conversion to thoracotomy	2 (20.0)
Thoracotomy	4 (40.0)
Postoperative complications	
Intraoperative blood transfusion	3 (23.1)
Pneumonia	2 (20.0)
Deep vein thrombosis	1 (10.0)

MPR, major pathological response; pCR, pathological complete response; VATS/RATS, video assisted thoracic surgery/robotic-assisted thoracic surgery.

Median operative duration was 240 min (range: 120–345 min), with the median estimated blood loss of 200 mL (range: 50–700 mL). The median postoperative hospital stay was 12.5 days (range: 5–20 days). Based on the Clavien-Dindo classification, 3 patients (3/10, 30.0%) experienced postoperative complications, including 2 cases of grade 1 pneumonia and 1 case of grade 3 deep vein thrombosis who was treated with inferior vena cava filter placement and anticoagulation therapy (Table 2).

### 3.4 TRAEs

All the patients experienced at least one grade 1–2 TRAE, with the most common being anorexia (70%) and alopecia (70%), followed by fatigue (60%). irAEs included grade 1 hypothyroidism (40%), grade 1 rash (20%), and grade 1 hepatitis (10%) (Table 3). All irAEs were managed per established guidelines. No severe irAEs, such as myocarditis, pneumonitis, or myasthenia gravis, were observed, and there were no treatment-related deaths.

**Table 3:** Treatment-related adverse reactions.

Adverse reactions, *n* (%)	Any grade	Grade 1	Grade 2
Chemotherapy-related adverse reactions			
Anorexia	7 (70.0)	7 (70.0)	
Vomiting	1 (10.0)	1 (10.0)	
Fatigue	6 (60.0)	6 (60.0)	
Leukopenia	2 (20.0)	1 (10.0)	1 (10.0)
Alopecia	7 (70.0)	7 (70.0)	
Arthralgia	3 (30.0)	3 (30.0)	
Limb numbness	3 (30.0)	2 (20.0)	1 (10.0)
Immune-related adverse reactions			
Rash	2 (20.0)	2 (20.0)	
Hepatitis	1 (10.0)	1 (10.0)	
Hypothyroidism	4 (40.0)	4 (40.0)	

### 3.5 Survival outcomes

**Figure 2:**
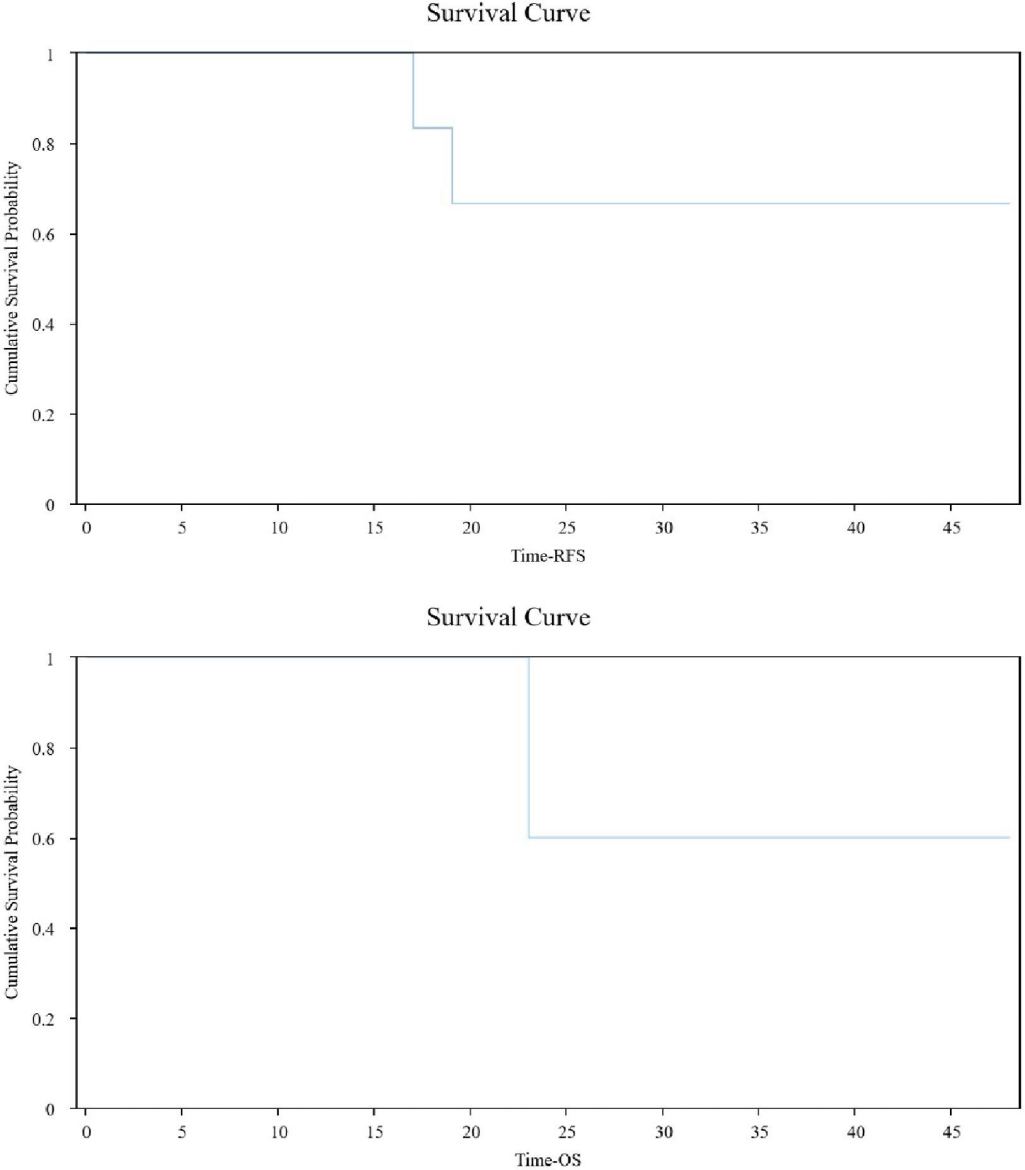
Analysis of survival outcomes in TSCC patients receiving perioperative immunotherapy.

The median follow-up duration was 30 months (range: 12–48 months). During this period, only 2 patients were dead, with RFS of 17 and 19 months and OS of 23 months for both, respectively. The median RFS (NA, 95% CI: 17.0-NA) and OS (NA, 95% CI: 23.0-NA) were both unreached (Figure 2). Notably, 2 patients who declined postoperative adjuvant treatment did not experience recurrence, suggesting a durable response to immunotherapy.

## 4 Discussion

The present study provides preliminary yet suggestive evidence regarding the clinical efficacy and safety of perioperative immunotherapy combined with chemotherapy in locally advanced TSCC. To the best of our knowledge, this is among the first studies focused on evaluating the integration of PD-1 inhibitors into perioperative management for TSCC. Our findings imply that addition of PD-1 inhibitors to perioperative treatment may potentially improve the DCR and R0 resection rates while maintaining a manageable safety profile, which may help address an important unmet need in the treatment of thymic carcinoma.

Unlike thymomas, thymic carcinomas rarely express autoimmune-related antigens (25, 26) but frequently exhibit moderate PD-L1 expression (27, 28) and tumor-infiltrating lymphocytes (TILs), suggesting a plausible biological basis for the application of ICIs (21, 22). In an open-label phase II trial using pembrolizumab monotherapy for refractory or relapsed thymic carcinoma, the ORR of 19.2% was reported, albeit in a palliative setting (22). In our study, the ORR and DCR were respectively up to 60% and 100% after neoadjuvant immunochemotherapy, which may be associated with the synergistic effect of ICIs combined with platinum-based chemotherapy, a strategy proven to enhance immunogenicity by promoting antigen release and dendritic cell activation (29). Notably, two patients in our study achieved pCR, consistent with the results of previous neoadjuvant studies in thymic carcinoma (30, 31). While pCR has not yet been formally defined for thymic carcinoma, the PR rate of 20% was observed in our cohort, suggesting that immunotherapy may hold promise for reshaping traditionally chemotherapy-dominated neoadjuvant landscape, even in less common malignancies such as thymic carcinoma.

Achieving R0 resection is paramount in thymic carcinoma, as incomplete resection correlates with dismal survival (4, 32, 33). In our cohort, 80% of patients underwent R0 resection after neoadjuvant immunochemotherapy, similar to the R0 resection rate of 77%–90% achieved with neoadjuvant chemoradiotherapy for thymic carcinoma (30, 31). The reduced tumor bulk and enhanced immune infiltration observed pathologically may reflect the dual mechanism of immunochemotherapy, namely cytotoxic chemotherapy makes the tumors reduced, while ICIs sustain anti-tumor immunity by blocking PD-1/PD-L1-mediated T-cell exhaustion. Notably, the lack of disease progression observed during neoadjuvant immunochemotherapy is particularly noteworthy, especially considering that rapid disease progression frequently hinders surgical intervention in locally advanced thymic carcinoma. These preliminary findings may offer insights that complement the conventional paradigm of upfront surgery for resectable tumors, suggesting that neoadjuvant immunochemotherapy could be a viable option to potentially optimize surgical outcomes.

Thymic carcinoma patients are uniquely vulnerable to irAEs due to the thymus's role in immune tolerance (21, 22, 34, 35). However, the patients in our cohort predominantly experienced grade 1 irAEs, such as hypothyroidism and rash, without grade 3–4 irAEs or treatment-related deaths. These outcomes may be attributable to the exclusion of patients with autoimmune comorbidities or paraneoplastic syndromes, which represents a common selection criterion in studies on thymic carcinoma (30). Despite prolonged use of PD-1 inhibitors (median 8.5 cycles), only one patient in our cohort discontinued treatment due to recurrence. It is worth noting, however, that continued vigilance for delayed irAEs remains important, given that survivors of thymic carcinoma often face long-term sequelae associated with multimodal therapy.

This study has several limitations that should be concerned. First, the small sample size and retrospective study design both limit the statistical power and the generalizability of the results. Second, all the patients in our study were diagnosed with stage III squamous cell carcinoma in histology, which precludes the efficacy assessment of immunotherapy in patients with other subtypes or stages. Third, as most patients remain alive, the statistical analysis of RFS and OS is currently precluded, and long-term survival outcomes require further investigation. Notably, the heterogeneity in adjuvant regimens, such as immunotherapy alone or immunotherapy combined with chemotherapy/radiation, complicates the outcome interpretation. Although consolidation strategies are often individualized in rare cancers, future studies should further standardize the adjuvant protocols to isolate the contribution of immunotherapy.

In conclusion, perioperative immunotherapy exhibits a promising resectable rate in locally advanced TSCC, with a manageable safety profile, although survival benefits have yet to be established. In the future, a prospective randomized controlled trial should be performed to further clarify the role of perioperative immunotherapy for locally advanced TSCC.

## Data Availability

The raw data supporting the conclusions of this article will be made available by the authors, without undue reservation.
